# Supplementary material for: Impact of HLA-DR Antigen Binding Cleft Rigidity on T Cell Recognition
Source: Int J Mol Sci. 2020 Sep 25;21(19):7081. doi: 10.3390/ijms21197081 (PMC7582474; doi:10.3390/ijms21197081)
Supplement: Supplementary file 1 [file ijms-21-07081-s001.pdf]

International Journal of *Molecular Sciences*

Supplementary materials

Impact of HLA-DR Antigen Binding Cleft Rigidity on T Cell Recognition

Christopher Szeto [1,†], Joseph I. Bloom [1,†], Hannah Sloane [1], Christian A. Lobos [1], James Fodor [1,2], Dhilshan Jayasinghe [1], Demetra S. M. Chatzileontiadou [1], Emma J. Grant [1], Ashley M. Buckle [1] and Stephanie Gras [1,3,*]

[1] Department of Biochemistry and Molecular Biology, Biomedicine Discovery Institute, Monash University, Clayton, VIC 3800, Australia; chris.szeto@monash.edu (C.S.); joseph.i.bloom@gmail.com (J.I.B.); hslo1@student.monash.edu (H.S.); christian.lobos@monash.edu (C.A.L.); fods12@gmail.com (J.F.); dhilshan.jayasinghe@monash.edu (D.J.); dimitra.chatzileontiadou@monash.edu (D.S.M.C.); emma.grant@monash.edu (E.J.G.); ashley@ptngconsulting.com (A.M.B.)

[2] Eccles Institute of Neuroscience, John Curtin School of Medical Research, The Australian National University, Canberra, ACT 0200, Australia

[3] Australian Research Council Centre of Excellence for Advanced Molecular Imaging, Monash University, Clayton, VIC 3800, Australia

* Correspondence: Stephanie.gras@monash.edu

† These authors contribution equally.

Residue	HLA-DR11	HLA-DR15	HLA-DR1
9	E	W	W
10	Y	Q	Q
11	S	P	L
12	T	K	K
13	S	R	F
26	F	F	L
28	D	D	E
30	Y	Y	C
31	F	F	I
37	Y	S	S
47	F	F	Y
58	E	A	A
67	F	I	L
70	D	Q	Q
71	R	A	R
96	H	Q	E
133	R	L	R
140	T	A	A
142	V	M	V
149	H	Q	Q

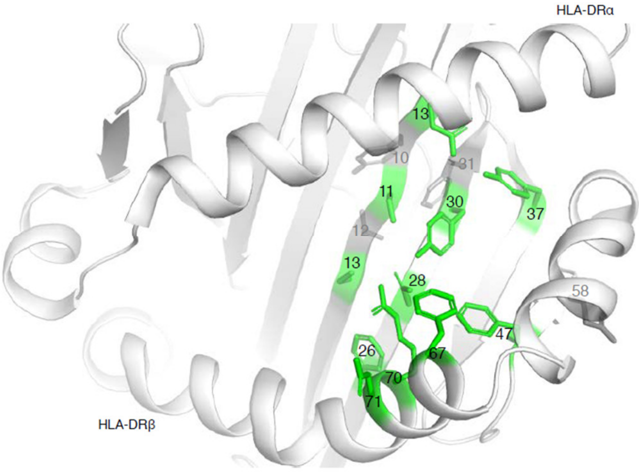

Figure S1. Polymorphic residues in HLA-DR antigen binding cleft. Crystal structure of HLA-DR11-RQ13 (PDB ID: 6CPN) (white) showing polymorphic residues (as sticks) that face towards (green) or face away (grey) from the antigen binding cleft. The table indicates the nature of the polymorphic residues among HLA-DR11, HLA-DR15 and HLA-DR1 molecules, and are coloured green if they are facing towards the peptide binding cleft.

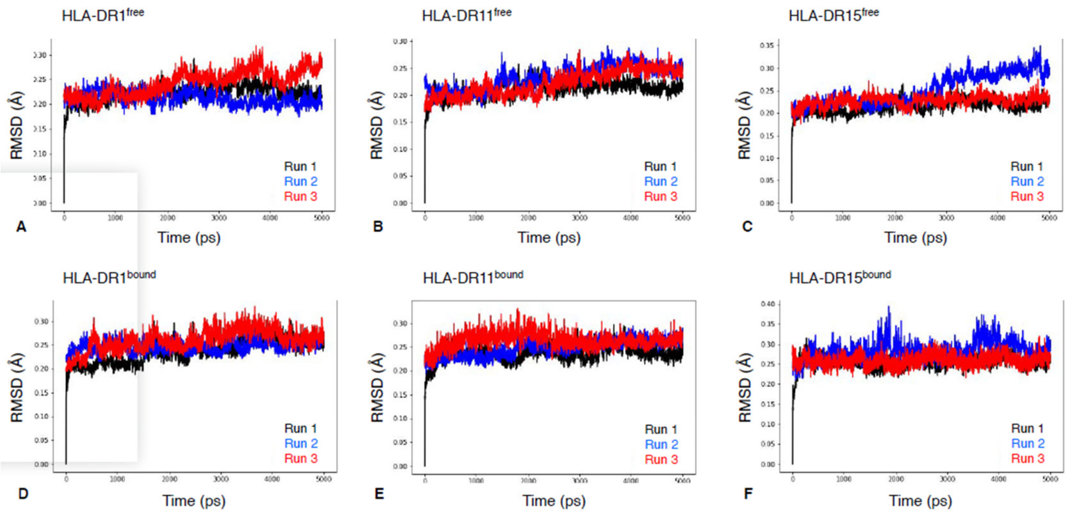

Figure S2. MD RMSD plots for HLA-DR molecules. MD RMSD plots for triplicate simulations over a 500 ns timeframe for HLA-DR molecules (**A**) HLA-DR1^{free}, (**B**) HLA-DR11^{free}, (**C**) HLA-DR15^{free}, (**D**) HLA-DR1^{bound}, (**E**) HLA-DR11^{bound}, and (**F**) HLA-DR15^{bound}. RMSD calculations are relative to the first frame of the first run.

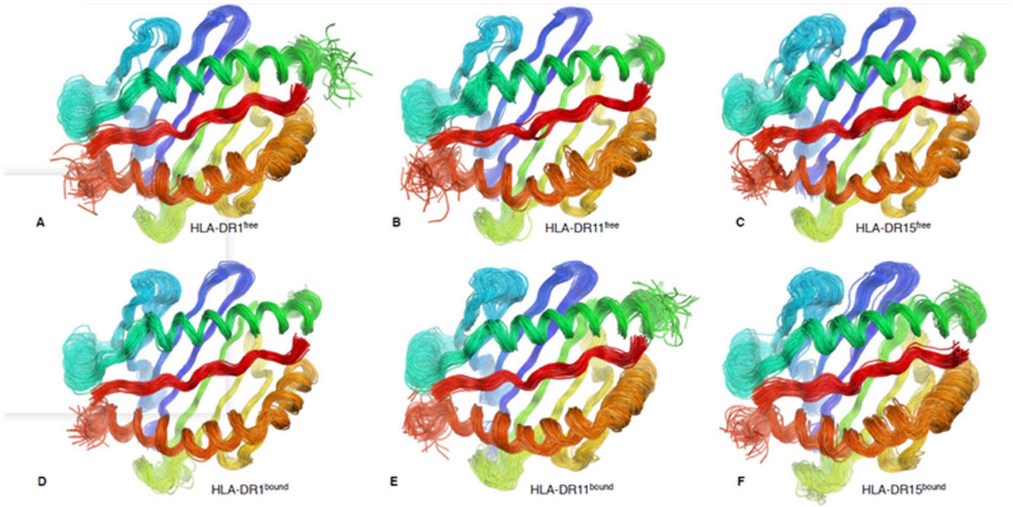

Figure S3. Structural overlay of MD simulations. MD structures taken from 500 ns triplicate simulations were overlayed for (**A**) HLA-DR1^{free}, (**B**) HLA-DR11^{free}, (**C**) HLA-DR15^{free}, (**D**) HLA-DR1^{bound}, (**E**) HLA-DR11^{bound}, and (**F**) HLA-DR15^{bound}. Each structure is a snapshot taken from MD trajectory at 20 ns intervals.

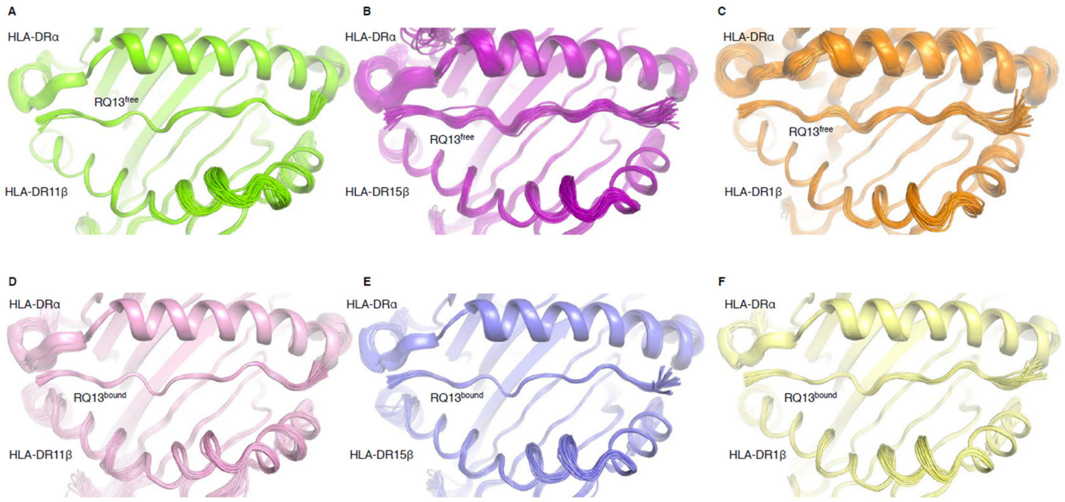

Figure S4. Overlay of ensemble models at the antigen binding cleft. Ensemble results of the antigen binding cleft (HLA-DR*α* represented by cartoon; HLA-DRβ and peptide as ribbon) for HLA-DR-RQ13 complexes in free (**A**–**C**) or F24 TCR bound (**D**–**F**) state. HLA-DR11-RQ13 structures are coloured in green (free, **A**) and pale pink (bound, **D**); HLA-DR15-RQ13 structures are coloured in purple (free, **B**) and blue (bound, **E**); and HLA-DR1 structures are coloured in orange (free, **C**) and yellow (bound, **F**).

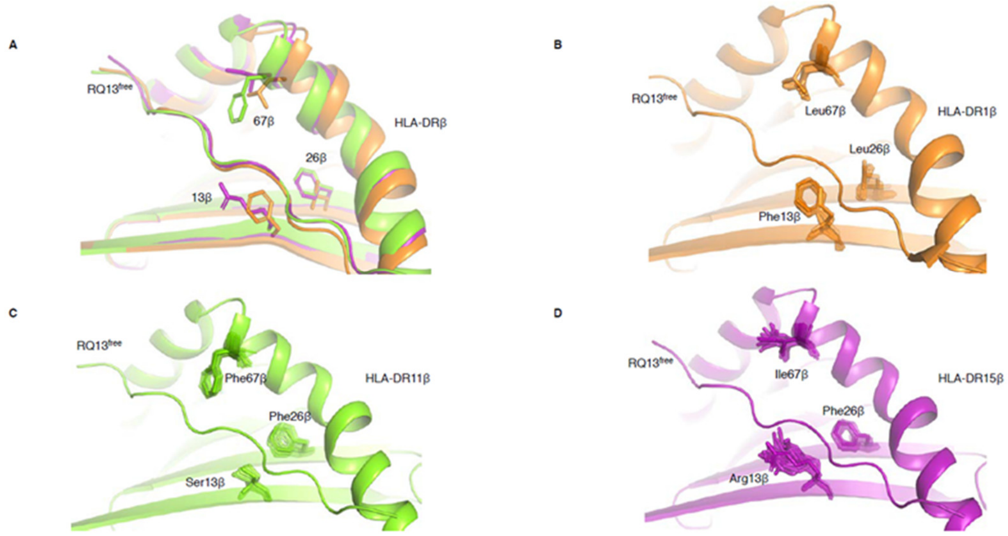

Figure S5. Conformational variation of polymorphic residues that open the cleft. **(A)** Structural alignment of crystal structures from HLA-DR-RQ13 complexes showing polymorphic residues HLA-DRβ13, HLA-DRβ26 and HLA-DRβ67 (stick) that cause the cleft to open. (**B** to **C**) Ensemble results of the same polymorphic residues for (**B**) HLA-DR11-RQ13 (**C**) HLA-DR15-RQ13 and (**D**) HLA-DR1-RQ13.

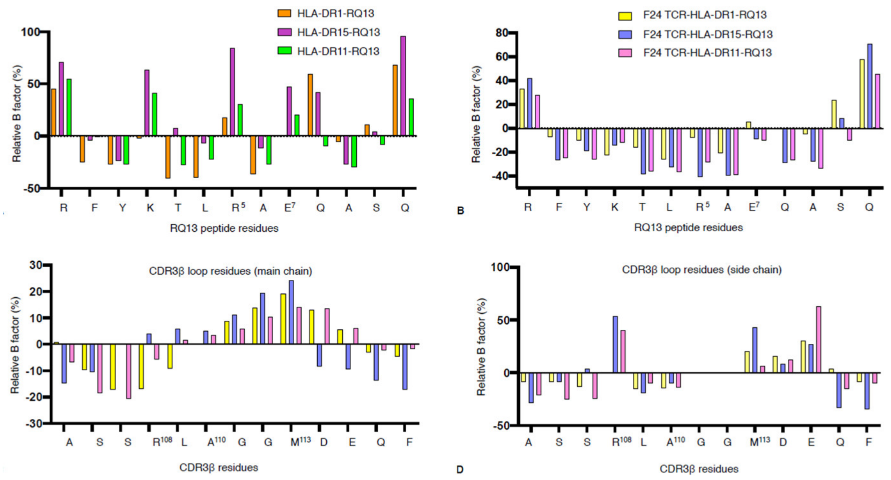

Figure S6. Relative B factor analysis of RQ13 peptide and CDR3β loop from the crystallographic structures. The relative B factor was calculated for the side chain of the RQ13 peptide residues from the HLA-DR-RQ13 structures in their free (**A**) and bound (**B**) states. The relative B factor (%) is calculated with the side chain average B factor $[100 \times ((B_{residue} - B_{cleft})/B_{cleft}))]$ and represented as coloured bar for each structures. (**A**) The bars are coloured in orange for HLA-DR1-RQ13, green for HLA-DR11-RQ13, and pink for HLA-DR15-RQ13 structures in their free state, or yellow, pale purple, pale pink in their bound state (**B**), respectively. For (**C**) and (**D**) panels, the relative B factor is calculated with the main chain (**C**) or side chain (**D**) of each residue of the F24 TCR CDR3β loop $[100 \times ((B_{residue} - B_{CDR3\beta})/B_{CDR3\beta}))]$.

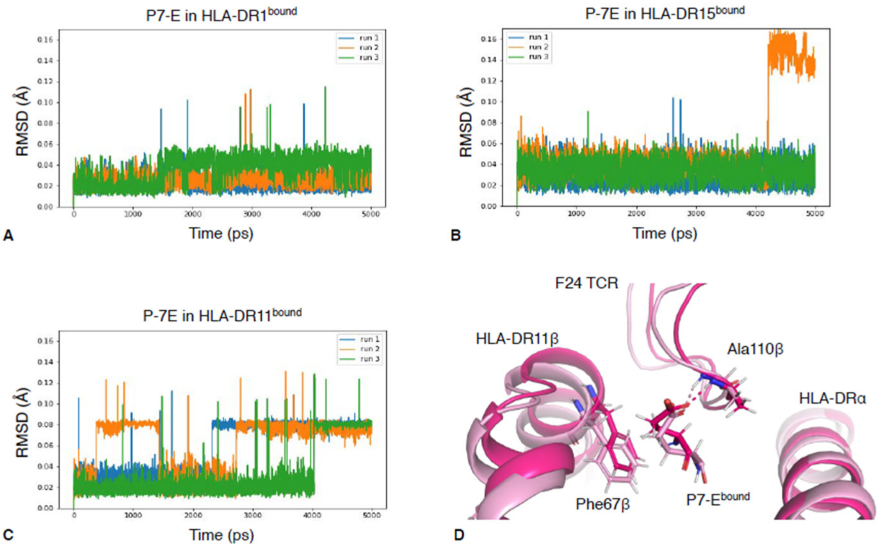

Figure S7. MD RMSD for P7-E side chain in HLA-DR^{bound} simulations. MD RMSD plots for triplicate simulations over a 500 ns timeframe for TCR-bound HLA-DR molecules for P7-E in (**A**) HLA-DR1, (**B**) HLA-DR15, (**C**) HLA-DR11. RMSD calculations are relative to the first frame of the first run. (**D**) Structural alignment of HLA-DR11^{bound} taken from Run 2 between frame 1500 (pink) and 4900 (dark pink) showing two structural conformations of P7-E forming a hydrogen bond with Ala110β (dashed line) with a bond distance of 2.2 Å and 1.8 Å, respectively.

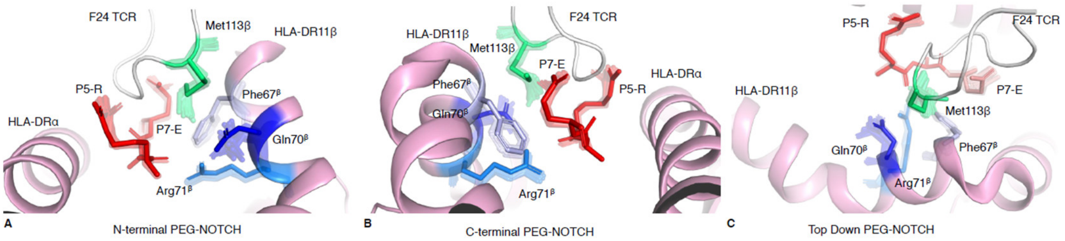

Figure S8. Spatial variation of residues that form the peg-notch interaction. The peg-notch interaction is driven by F24 TCRβ loop (white) Met113β (turquoise) that inserts itself into the notch formed by HLA-DR11-RQ13 (light pink). Each panel shows the same peg-notch interaction from the point of view of (**A**) the N-terminus (**B**) the C-terminus and (**C**) a top-down TCR perspective. The RQ13 peptide residues at position 5 and 7 are represented as red sticks, while HLA-DR11 residue Phe67β, Gln70β and Arg71β are represented as blue sticks.

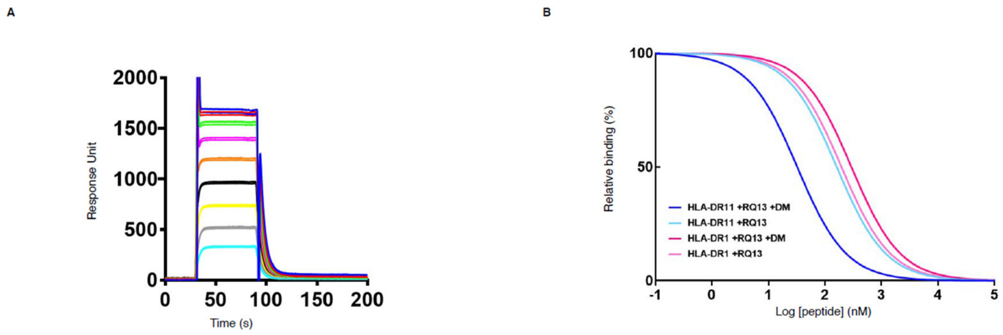

Figure S9. SPR and peptide binding exchange assays. (**A**) F24 TCR binding kinetics to HLA-DR1-D66A$^{\beta}$ mutant presenting RQ13 peptide determined by SPR. The curves represent the concentration range used of F24 TCR. (**B**) The displacement of HA$_{TAMRA}$ peptide by increasing concentrations of RQ13 peptide. The shift in binding curves of HLA-DR11 (light blue) indicates that the addition of HLA-DM facilitates displacement of HA$_{TAMRA}$ by RQ13 peptide (blue), whereas, HLA-DR1 (light pink) and HLA-DR1 in the presence of HLA-DM (pink) shows no shift.

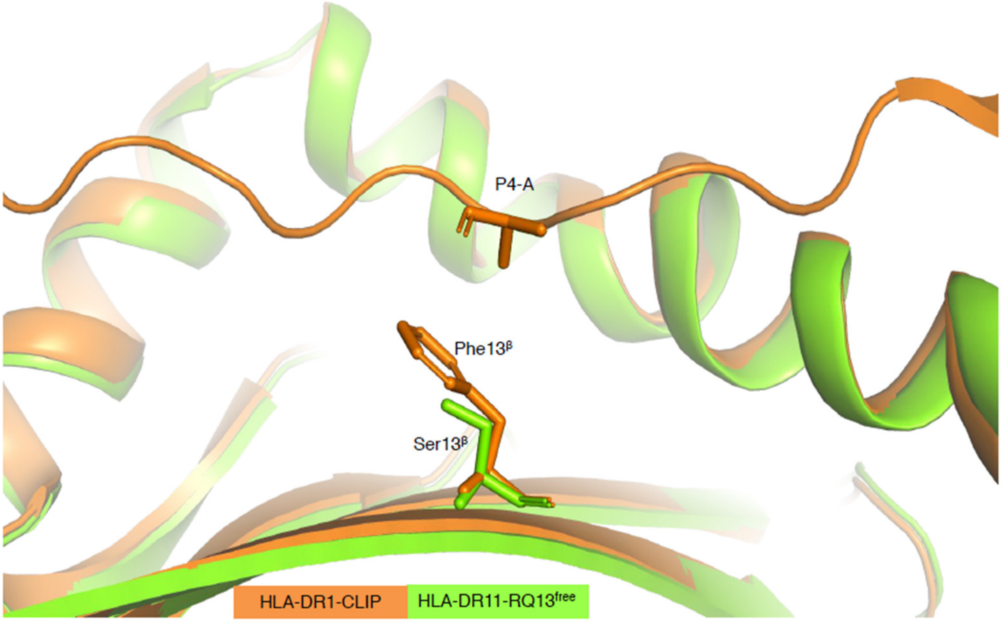

Figure S10. Structural alignment of HLA-DR11-RQ13 and HLA-DR1-CLIP. Crystal structure of HLA-DR1-CLIP (PDB ID: 3PDO, orange) shows that polymorphic residue Phe13β can form VdW interactions with P4-A in HLA-DR1-CLIP, but this may not be possible for Ser13β in HLA-DR11 (green).
